# Why *are* patients with chronic myeloid leukaemia (non-)adherent?

**DOI:** 10.1038/bjc.2012.349

**Published:** 2012-09-04

**Authors:** I Abraham, K MacDonald

**Affiliations:** 1 Center for Health Outcomes and PharmacoEconomic Research, College of Pharmacy, University of Arizona, Drachman B-211G, 1295 N Martin, Tucson, AZ 85721, USA; 2 Matrix45, Tucson, AZ 85743, USA

Until the advent of imatinib as an alternative to interferon alfa, chronic myeloid leukaemia (CML) was a lethal disease with a life expectancy post diagnosis of a few years. Imatinib, and other tyrosine kinase inhibitors since, have transformed CML from a lethal disease into a chronic illness. For instance, among patients in the pivotal IRIS study (O’Brien *et al*, 2003), durable responses have been observed 5–8 years out (Druker *et al*, 2006; Hochhaus *et al*, 2008; Deininger *et al*, 2009). Patients in complete cytogenetic response (CCyR) after 2 years (±3 months) have the same life expectancy as the general population (Gambacorti-Passerini *et al*, 2011). This requires long-term, if not life-long, imatinib treatment – and the expectation that patients be adherent and take their medication as agreed upon with their clinician.

If patients with a heretofore lethal disease now have access to a highly efficacious targeted oral treatment with a modest if not benign side-effect profile, could (and should) we not expect them to be adherent? Unfortunately, as we first learned from the ADAGIO study, the assumption of a rational CML patient may not hold (Noens *et al*, 2009): about one-third of the 169 patients were classified as non-adherent per brief structured patient interview. Ninety-day manual pill counts averaged 90.9% of pills taken to pills prescribed. Using electronic event monitoring to determine the percentage of dose taken to prescribed, a median observation period of 91 days, and non-adherence criterion of ⩽90%, the Hammersmith group classified about a quarter of patients as non-adherent (Marin *et al*, 2010) – a rate also observed by Ganesan *et al* (2011), who used an unwarranted interruption of treatment >1 week as criterion. These are not very encouraging results as they exceed the non-adherence rate of 20.9% reported in a meta-analysis of 65 studies involving cancer patients (DiMatteo, 2004a). The imatinib non-adherence news may get even more discouraging, if we consider the study by Efficace *et al* (2012) in this issue of the Journal. Using a similar but different structured patient interview, 194 of 413 patients were classified as non-adherent; statistically, that is one out of two patients. Granted, recently a single-centre study of near-perfect adherence in 37 out of 38 (97.4%) imatinib-treated CML patients was published (Jönsson *et al*, 2012). However, these patients were interviewed by phone, not in the clinic. The high likelihood of social desirability bias, especially in a phone interview with an independent research nurse, and in the absence of the full range of verbal and non-verbal data from live clinical assessment, is a cause to dismiss the findings of this study as, well, singular.

It might indeed be a good time to take stock of what is known, what is not known, and what still needs to be known about adherence to imatinib – and thus contribute insights into the adherence to oral antineoplastic treatment generally (Partridge *et al*, 2002; Ruddy *et al*, 2009). With non-adherence to imatinib, the concerns are: what factors have been identified that may either enhance adherence or engender non-adherence behaviour? and how do Efficace *et al* (2012) advance what is known?

Using a qualitative approach, the Hammersmith group derived a helpful framework for classifying the reasons for non-adherence *vs* adherence (Eliasson *et al*, 2011). Among others, some reasons concerned unintentional non-adherence, for instance, forgetting to take, accidentally taking too much, or no supply at the pharmacy. Others were intentionally non-adherent owing to side effects, social activities, travel, or changes in dosing. Some patients may have underestimated the consequences of non-adherence: perceived consequences, awareness of clinicians’ desire for adherence yet also lack of communication, reliance on monitoring, and healthcare providers to detect and relay changes in clinical parameters, not believing that missing an odd dose would make a difference, and seeing positive reinforcement in prior non-adherence episodes. In contrast, reasons for being adherent included the relative absence of side effects, faith in healthcare team and imatinib, and understanding illness and treatment. A potential mismatch in communication between clinicians and patients was cited. In a separate quantitative analysis, the Hammersmith group identified younger age, adverse effects, and unexplained increases in BCR-ABL1 transcript levels as being associated with non-adherence (Marin *et al*, 2010).

Although some of these factors were also identified in ADAGIO study, which was, by lack of a better word, more traditional in its quantitative examination of determinants (Noens *et al*, 2009). Non-adherence was found to be a function of patient demographics (male, younger, living alone), disease and treatment variables (longer time since diagnosis of CML or start of imatinib therapy, higher than standard imatinib dosing), poor functional status, and negative perceptions about the quality of chronic care. Non-adherence was more likely among patients of physicians who had been practicing longer; and, seemingly paradoxical but perhaps indicative of physician concern about poor treatment response, longer duration of treatment follow-up visits. In contrast, adherence was a function of patients’ knowledge of disease and treatment, number of daily medications, achieving secondary education, and medication behaviour self-efficacy. Physician-related variables also had a role: more patients with CML in one’s patient panel, more time spent in the first visit with newly diagnosed patients, working in a teaching hospital, and being a haematologist as opposed to an omnipracticing cancer physician.

Both the ADAGIO and Hammersmith studies may have hinted at some determinants to which the Efficace *et al* (2012) study now provides answers and new insights. The Hammersmith group noted a mismatch in communication between clinicians and patients (Eliasson *et al*, 2011). Patients seemed not to believe in a link between their non-adherence and their treatment response – looking instead to their clinicians to tell them if there was a relationship between medication behaviour and treatment outcome. Clinicians, on the other hand, tended to give patients positive feedback on their clinical response, while often being unaware of any adherence problems. Efficace *et al* (2012) noted that patients were more likely to be adherent if they were satisfied with the information clinicians gave them regarding their disease, the side effects of therapy, and the impact of disease and side effects on quality-of-life. The latter may be the most important, as it translates the rather static factor of knowledge about disease and treatment knowledge into patients’ perceived impact of both on their daily life and well-being. If we are to better understand why some patients are (non-)adherent, it is imperative to examine their ability to integrate disease and treatment into their daily life, the priorities they like to preserve, the compromises they are willing to make, and the psychological, social, and physical impact of adapting to disease and treatment. In other words, the Efficace *et al* (2012) findings advise us not to merely provide clinical information to patients, but to take the time to explore the impact of disease and treatment on patients’ quality-of-life, how they cope with the burden, and how they value the remission of their CML in daily life.

Perhaps hidden in patients’ desire for more information may be the wish to hear from their clinicians ‘how much they can be non-adherent’. The answer should be that this is not yet known – and that, in the interim, patients should be highly if not perfectly adherent. However, the latitude for non-adherence to imatinib needs to be assessed, specifically if a threshold, tipping point, or tipping range exists below which risk of impaired response accelerates. Such information would enable clinicians to educate patients in clear and concrete terms with regard to the risks and consequences of non-adherence. We would hypothesise that the margin for non-adherence would be small, as we have learned from heart transplantation where immunosuppression treatment failure results in organ rejection. In a study of 101 transplant patients at a median of 3 years post-transplant, De Geest *et al* (1998) found that despite the omnipresent threat of rejection, 7% of patients were minor and 9% moderate subclinical non-compliers. Subclinical non-compliance was associated with late acute rejection episodes: 1.19% among compliers, 14.28% in minor non-compliers and 22.22% in moderate non-compliers.

Rather curiously, the ADAGIO study found that the demographic variable of living arrangement and living alone in particular was related quite strongly to non-adherence. Efficace *et al* (2012) included a patient-reported questionnaire focused on the functional aspects of social support and measuring the strength and quality of patients’ relationships with family, friends, and significant others. Functional social support indeed proved to be a predictor of adherence. As the authors explain, patients with stronger social networks may be more likely to be reminded to take their medications. Citing DiMatteo (2004b), the authors also advise that social support may promote patients’ coping with the burden of lifelong therapy. This finding, too, has important clinical implications – perhaps not fully realized by Efficace *et al* (2012): engage at least part of the social network (e.g., spouse, child, friend,…) in the disease and treatment process, for instance, by involving them in clinical visits and treatment, counselling them about the critical importance of adherence, and educating them how they can promote adherence in their loved one.

Counter to the common observation that polypharmacy is associated with a greater risk for non-adherence, Efficace *et al* (2012) echoed the ADAGIO findings that a higher medication burden was associated with a greater likelihood of adherence. Although the measures were different between the two studies, several explanations may be plausible. For example, patients who are already on a medication regimen may be less likely to forget to take their imatinib; or patients who have comorbid conditions requiring drug therapy may be more concerned about their health, fearful of the risk of relapse, knowledgeable about their diseases and treatments, or have more frequent contact and communication with their physicians, pharmacists, and nurses.

While for most patients imatinib has relegated CML to a manageable chronic disease, this may prove to be a double-edged sword. There no longer is the dire diagnosis, shortened life expectancy, and high mortality. Patients and their clinicians begin treatment with the belief that remission is assured. Yet, over time, they may be lulled into the complacency of chronicity seen in long-term illness in general – among patients, lack of symptoms (‘I don’t feel sick anymore’), fatigue with treatment side effects, or the false belief that small variations in adherence might not have significant consequences; among physicians, therapeutic inertia, failure to assess for medication non-adherence, or lack of (time for) adequate patient education.

Together, the ADAGIO, Hammersmith, and Efficace *et al* (2012) studies are providing cancer physicians, nurses, and other staff with significant insights. Frankly, after these three studies, those caring for patients with CML no longer have an excuse not to evaluate medication behaviour, not to prevent or correct non-adherence, and not to promote adherence as part of routine clinical practice. The effect of non-adherence on treatment response has been documented at the levels of cytogenetic (Noens *et al*, 2009; Ibrahim *et al*, 2011) and molecular response (Marin *et al*, 2010) as well as event-free survival (Ganesan *et al*, 2011). The risk is just too great as we are reminded that ‘poor adherence is the main reason for loss of CCyR’ (Ibrahim *et al*, 2011) and ‘adherence is the critical factor for achieving molecular responses’ (Marin *et al*, 2010). Whether there is any latitude for non-adherence is unknown. In the best interest of patients, it should not be assumed.
